# The Social Representations of Pornography Consumers Among Individuals in Romantic Relationships: Exploring the Roles of Gender, Relationship Satisfaction, and Sexual Satisfaction in a Romanian Sample

**DOI:** 10.1007/s10508-024-03025-x

**Published:** 2024-11-08

**Authors:** Tudor-Daniel Huțul, Andreea Huțul, Andrei Corneliu Holman

**Affiliations:** 1 Sapienza University of Rome, Rome, Italy; 2 Faculty of Psychology and Education Sciences, Alexandru Ioan Cuza University of Iași, 700554 Iași, Romania

**Keywords:** Social representation, Pornography, Relationship satisfaction, Sexual satisfaction, Gender

## Abstract

Understanding the psychological conflicts associated with pornography consumption can help professionals tailor their interventions to address the mental health risks faced by individuals struggling with issues related to their pornography use. The main objective of this research was to investigate how pornography consumers are perceived from a social representation theoretical perspective and to examine variations in these perceptions—or social representations—according to gender, relationship satisfaction, and sexual satisfaction. All participants (N = 875 Romanians) were in a romantic relationship at the time of participation. Our results indicated that the social representation of pornography consumers varied significantly depending on gender, sexual satisfaction, and relationship satisfaction. Also, our findings showed a potential psychological conflict in the long run, which can put individuals at risk for self-stigma, guilt, and subsequent mental health consequences. We discuss conclusions from both a theoretical and practical perspective, with a focus on the utility of our conclusions in the clinical practice context of psychologists, psychotherapists, and mental health workers when dealing with issues related to pornography use.

## Introduction

For more than 25 years, the increased accessibility of pornography afforded by the internet has led to an increase in research on the topic (Grubbs et al., 2020). Various definitions of pornography have emerged in the literature in recent years, with one describing it as any material, text, visual, or video content created with the intention of sexually arousing the user (Willoughby & Dover, 2023). When this online content is used regularly, it can lead to problematic consumption, which includes aspects like self-perceived addiction, inappropriate use of pornography in contexts such as work, school, or university, or its utilization as maladaptive coping with negative emotions (Bőthe et al., 2019; Grubbs & Perry, 2019; Huțul, 2023; Karner-Huțuleac & Huțul, 2023; Kor et al., 2014; Kraus et al., 2015).

The research conducted over the years has shown that the impact of pornography varies based on the context of each individual, potentially leading to both positive and negative effects. Among the positive or neutral effects associated of pornography consumption, physical, sexual satisfaction or intimate relationship satisfaction has been identified in the literature (Dwulit & Rzymski, 2019; Grubbs & Gola, 2019; Kohut et al., 2017; Vaillancourt-Morel et al., 2019), while other studies highlighted associations with negative dynamics pertaining to couple relationships, social connections, well-being, and even brain functions (Gola & Draps, 2018; Gola et al., 2016, 2017; Kowalewska et al., 2018; Lambert et al., 2012; Lewczuk et al., 2017; Love et al., 2015; Potenza et al., 2017; Sun et al., 2016; Voon et al., 2014). This prior research indicates that the potential effects related to pornography consumption likely depend on an array of factors. The purpose of this study is to examine the social representation of pornography users as one of these important factors.

### Theory of Social Representations

The term “social representation” was first coined by Moscovici (1961), and this concept has a history of over 60 years. Social representations (SRs) are defined as a “system of values, ideas, and practices with a twofold function; first, to establish an order which will enable individuals to orient themselves in their material and social world and to master it; and secondly to enable communication to take place among the members of a community by providing them with a code for social exchange and a code for naming and classifying unambiguously the various aspects of their world and their individual and group history" (Moscovici, 1973). Generally, the theory of social representations (SRT; Moscovici, 1961, 1987, 1988, 2001) is concerned with how individuals produce meaning, and particularly common sense, in their daily lives by examining the opinions, knowledge, and beliefs that revolve around particular social phenomena (Moscovici, 1988, 2001). Furthermore, the study of social representations provides valuable information for understanding the motivations behind decisions and human behavior (Rateau et al., 2012), and SRT has been effectively utilized to explore the collective comprehension of a social phenomenon and the associated behaviors within groups or communities in various countries (e.g., Ahn & Jung, 2016), including in Romania (e.g., Arhiri et al., 2022; de Rosa & Holman, 2011; Holman et al., 2010; Todeancă et al., 2019).

SRT (Moscovici, 1961, 1987, 1988, 2001) may also be related to the negative effects associated with pornography use. The more taboo a practice, is—such as pornography use—and the more the practice is socially stigmatized, the greater the distress for consumers can be. A stigmatizing SR can foster consumers’ belief that engaging in pornography use is morally wrong and goes against societal norms, which can lead to feelings of guilt and moral conflict (Grubbs et al., 2019). These negative feelings can bring psychological and psychosocial distress and are linked to moral incongruence over pornography use (Grubbs et al., 2019).

### Stigma, Pornography Use, and Social Representations

Stigma is regarded as a sign of shame, disapproval, or disagreement that leads individuals to experience outcomes such as rejection, discrimination, or even exclusion from various activities in different domains of society (Guilbert, 2002). The stigma associated with pornography and its consumers has developed over several decades. For example, in 1980, it was stated that “if pornography does not offend local community standards, we say, then something is wrong because it should” (Kipnis, 2007). This indicates that the understanding of behaviors associated with pornography consumption is not derived from specific and measurable characteristics, but rather from how these behaviors are perceived as objectionable or transgressive (Macleod, 2021). In this context, pornography has been linked to transgression and has acquired a taboo connotation (Hester, 2014). Despite the passage of several decades since pornography was condemned in the harshest terms and the changes it has undergone over time, particularly due to the rapid evolution of the internet and digital technologies, it continues to be associated with stigma and shame (Macleod, 2021; Voss, 2015).

This societal stigma of pornography can generate self-stigma among pornography consumers, which represents the internalization of aspects such as prejudices, stereotypes, or negative beliefs regarding an individual’s characteristics (Fan et al., 2022). Generally, self-stigma can bring about health and social problems (Chang et al., 2020, 2022), depression and demoralization (Ritsher & Phelan, 2004; Ritsher et al., 2003), a low self-esteem (Lysaker et al., 2006, 2008), or social avoidance (Ritsher et al., 2003; Yanos et al., 2008). Past research indicated that one's stigmatized status as a pornography consumer is associated to negative mental health outcomes, such as feelings of depression and despair in relation to the use of pornography (Tewksbury, 2012). It may also negatively affect individuals' relationships (Grov et al., 2011; Maddox et al., 2011; Olmstead et al., 2013; Perry, 2016, 2017a; Willoughby et al., 2016). Furthermore, a systematic review on the links between pornography consumption and sexual functioning, including sexual satisfaction, found that poorer sexual functioning was less frequently related to pornography use itself, but more consistently to perceived problematic pornography use (Hoagland & Grubbs, 2021). This supports the assertion that pornography users’ convictions that their behavior is morally or socially repulsive, grounded in negative societal representations, may exacerbate their distress.

An important role in these psychological dynamics is played by the fact that the consumption of explicit sexual material breaches religious-moral norms sanctioning such behavior. As such, religiosity is strongly associated with a negative perception of pornography viewing, which is driven by the moral values held by individuals (Grubbs et al., 2015; Huțul & Karner-Huțuleac, 2023a, 2024a; Picone, 2016; Thomas, 2013; Volk et al., 2016). This condemnation of pornography based on religious grounds can lead to self-stigma among those who watch it due to feelings of shame or scrupulosity, causing them to distance themselves from family members or friends (Short et al., 2012) and thus further enhancing the risks of adverse mental health consequences.

Overall, these findings indicate that stigmatizing SRs may play a significant role in pornography users' interpersonal and intrapersonal distress. Furthermore, they suggest that pornography users may be strongly stigmatized in Romania, where the Christian Orthodox religion, which is shared by almost 74% of the population according to the 2022 census (Romanian National Institute of Statistics, 2024), has played a crucial role in shaping the political and social life of Romanian citizens, as well as their moral values (Turcescu & Stan, 2005). The Romanian Orthodox Church has strived, since the fall of communism, to impose its own ideas regarding sexuality and to define what constitutes acceptable and unacceptable sexual behavior from a religious perspective (Turcescu & Stan, 2005). Therefore, pornography is often considered morally condemnable and taboo in Romania (Gergely & Rusu, 2023; Huțul & Karner-Huțuleac, 2023a, 2024a), as society as a whole rejects any discussion related to sexuality and its associated components.

Given our emphasis on how SRs (Moscovici, 1961, 1987, 1988, 2001) have the potential to engender psychological conflicts, it is important to address the “Pornography Problems Due to Moral Incongruence” (PPMI; Grubbs et al., 2019) model, which represents one of the most important explanatory frameworks of problematic pornography consumption (Borgogna et al., 2023). The model highlights that issues regarding pornography use may stem from two different pathways, as follows: (I) “pornography problems due to dysregulation,” and (II) “pornography problems due to moral incongruence” (Grubbs et al., 2019). Prior to the emergence of this theoretical model, aspects related to moral cognitions and morality-related constructs were in the best case neglected. The PPMI model highlights the key role that moral incongruence plays in the psychological conflict that pornography use can generate (Grubbs et al., 2019). Moral incongruence represents the difference between one's own beliefs regarding the moral disapproval of pornography and one’s actual pornography use (Grubbs & Perry, 2019; Ostrander, 2021). While this difference has been primarily discussed in the PPMI model examining religiosity as a predictor, other studies highlighted that the moral disapproval of pornography cannot solely originate from religiosity (Vaillancourt-Morel & Bergeron, 2019). Negative SRs may also constitute determinants of one’s moral incongruence. Specifically, as long as society itself disapproves of pornography, considering it taboo (Gergely & Rusu, 2023; Huțul & Karner-Huțuleac, 2023a, 2024a) and fundamentally wrong, pornography users’ moral beliefs may align with these negative SRs, thus generating self-stigma in these individuals.

### Pornography Use, Relationship Satisfaction, and Sexual Satisfaction

The way people perceive pornography use may be related to relationship satisfaction and/or sexual satisfaction. Regarding sexual satisfaction, it is one of the most vital expressions that an individual can have concerning sexual health (WHO, 2010a). This standpoint is universally applicable to people, including middle-aged or older adults (Buczak-Stec et al., 2023). Also, sexual satisfaction plays a significant role in various aspects of life, from people’s physical and emotional health to the general well-being of families (WHO, 2010b). When individuals have an unsatisfactory sexual life, pornography represents one of the few sources through which sexual satisfaction can be achieved (Daspe et al., 2018; Olmstead et al., 2013). However, equally important is the research finding that the use of pornography is associated with lower sexual satisfaction (Doran & Price, 2014; Morgan, 2011; Peter & Valkenburg, 2009; Wright et al., 2018; Zillmann & Bryant, 1988). Also, the consumption of pornography has been found to be negatively associated with couple satisfaction (Bridges & Morokoff, 2011; Campbell & Kohut, 2017; Maddox et al., 2011; Muusses et al., 2015; Perry, 2017b; Peter & Valkenburg, 2009). This relationship stems from the fact that the use of pornography can sometimes be employed as a means of disconnecting from an unsatisfactory sexual life (Peter & Valkenburg, 2010). Therefore, we expect that this higher frequency of pornography use among individuals with low relationship and sexual satisfaction to be associated with a less negative and incriminatory SR and more aware or the potential benefits of pornography in offering ways to cope with the challenges of an unsatisfactory couple relationship.

Another relevant aspect to be considered is that men consume much more pornography than women (Huțul & Karner-Huțuleac, 2023a; Komlenac & Hochleitner, 2022; Landripet & Štulhofer, 2015; Miller et al., 2019; Sun et al., 2016). This could motivate the normalization of pornography use among men in comparison to a more deviance–focus representation among women. Secondly, women may have a different SR compared to men due to the fact that for them pornography use may be seen as a source of dissatisfaction within the couple relationship, diminishing attraction toward the primary partner (Stewart & Szymanski, 2012). Moreover, concerning women, various studies suggest that partners' pornography consumption can generate feelings of jealousy and overall potentially erode the quality of the relationship (Bergner & Bridges, 2002; Daneback et al., 2009; Grov et al., 2011; Perry & Davis, 2017; Schneider, 2000; Stewart & Szymanski, 2012; Zitzman & Butler, 2009). These findings suggest further factors of a more negative SR of pornography among women in comparison to men, as the former are more concerned about the negative effects that pornography consumption may have on the primary relationship and experience more distress toward this behavior.

### The Present Study

The aim of the current study was to investigate the content of the SR of the “pornography consumer” within a sample of Romanians and to examine its variations according to individuals' relationship satisfaction, sexual satisfaction, and gender. As the studies reviewed above indicate, negative SRs and their associated stigma have the potential to inflict adverse psychological outcomes among individuals who engage in pornography consumption. Furthermore, past research highlighted significant associations between the individual’s pornography consumption and negative dynamics in his/her romantic relationship. By highlighting the specific patterns of socially representing pornography consumption in the distinct groups generated by the factors considered (i.e., gender and relationship/sexual satisfaction), this study can contribute to the extant knowledge on the different meanings that people attach to pornography and on the potential outcomes of its consumption. Besides this exploratory aim and based on the findings outlined above, our research addresses the hypothesis that women and individuals with low relationship and sexual satisfaction have more negative SRs of pornography consumers than their counterparts, i.e., men and individuals with high relationship and sexual satisfaction, respectively.

## Method

### Participants and Procedure

Eight hundred seventy-five individuals, involved in romantic relationships, participated in this study, ranging in age from 18 to 69 years (M = 30.25; SD = 10.27). Among these, 639 were females (72.6%), and 239 were males (27.4%). In terms of marital status, 331 individuals (37.8%) were married at the time of completing the battery of instruments, while 544 (62.2%) were in a couple relationship but unmarried.

This study utilized the snowballing technique with convenience sampling, and the research questions were addressed through the Google Forms online platform. The questionnaire link was disseminated on various social media groups in the major cities of Romania (e.g., groups related to residence in Bucharest, Iași, Cluj, or Galați). Before completing our battery of instruments, each individual was required to read the informed consent. It specified that participation is 100% voluntary, respondents will not be rewarded in any way for their participation, they can withdraw from the study at any time without consequences, and the data will be used exclusively for academic purposes, ensuring confidentiality. Additionally, participants were provided with an email and a phone number for additional questions or assistance, if needed. The average completion time was approximately 7 min.

In terms of eligibility criteria, the criteria selected were as follows: (1) a minimum age of 18 at the time of completing the survey—i.e., the legally recognized age in Romania, and (2) being in a couple relationship at the time of completing the questionnaires, regardless of whether it was a marriage or a non-marital romantic relationship.

### Measures

Before proceeding with our questions regarding the social representation of pornography consumers in Romania, we provided the following definition to our respondents: “Pornography refers to any sexually explicit films, video clips, or pictures displaying the genital area, with the intention to sexually arouse the viewer; this may be seen on the internet, in a magazine, in a book, or on television” (Kohut et al., 2020). We chose to use this definition because it is one of the most widely employed in research on pornography (Grubbs et al., 2022), including in studies conducted on samples from Romania (Huțul & Karner-Huțuleac, 2023b; Huțul et al., 2024).

**Social representation of pornography consumer.** We used a free association task in which participants were asked to write the first, second, and third word or phrase they associated with the concept of “pornography consumer.” This method is based on the “Associative Network” instrument (De Rosa, 2002) and has also demonstrated its validity in other previous studies regarding the content of social representation (de Rosa & Holman, 2011; Gherman et al., 2022; Holman et al., 2010). The words and expressions extracted from the participants' answers were submitted to a qualitative analysis. Two independent researchers analyzed the textual data inductively and developed in vivo codes, by using, as much as possible, the words elicited from participants to label each code (Saldaña, 2021). Synonyms and, generally, words or expressions similar in meaning were grouped in the same code or lexical element, while also considering, where applicable, the semantic context offered by the respective expressions. For instance, obscenity, someone who enjoys vulgar things and profanity were grouped in the same code (“obscenity”), “sick,” “sickness,” “suffering from an illness,” unwell person in “sick,” stupidity, idiotic, feeble-minded, not able to think straight in “stupidity.” This process also involved, in instances where a word yielded multiple forms across the textual data, the lemmatization of lexical elements, reducing each word to its most common form in everyday language. Disagreement between coders appeared in less than 10% of the cases, and in these situations consensus on the final code was reached through discussions.

**Relationship satisfaction.** To measure relationship satisfaction, we employed a single item (“In general, how satisfied are you with your relationship?”). This item is the sole component of the Relationship Assessment Scale-1 (RAS-1; Fülöp et al., 2022), the shortened version of the Relationship Assessment Scale-7 (RAS-7; Hendrick, 1988), and it was assessed using a 7-point Likert scale, where 1 corresponded to “Extremely unsatisfied,” and 7 to “Extremely satisfied.” The use of this item has demonstrated its psychometric properties (Fülöp et al., 2022). High scores indicate a high level of relationship satisfaction.

**Sexual satisfaction.** To assess individuals' sexual satisfaction, a single item was employed “With respect to the past month, to what extent are you satisfied with your sexual life in general.” This item was assessed using a 7-point Likert scale, where 1 corresponded to “Extremely unsatisfied,” and 6 to “Extremely satisfied.” This has been utilized in other recent works (Daspe et al., 2018), including studies that have involved the Romanian population in the context of pornography use (Huțul & Karner-Huțuleac, 2024b), and the validity of a single item for assessing sexual satisfaction has been previously shown (Mark et al., 2014). The responses provided were reversed. Higher scores indicate a higher level of sexual satisfaction among the individuals.

**Sociodemographic variables.** Individuals reported their gender, age, rural or urban background, marital status, and the length of their relationship.

To ensure the preservation of the meaning of all instruments involved, we translated their items using the backward method, incorporating all recommendations from the literature regarding the translation and adaptation of instruments (Beaton et al., 2000; Hambleton & Zenisky, 2010; Maneesriwongul & Dixon, 2004).

## Results

The descriptive statistics for relationship and sexual satisfaction are presented in Table 1. First, participants were divided into two categories based on relationship satisfaction, using the median of the distribution as reference point: (1) individuals with low relationship satisfaction and (2) individuals with high relationship satisfaction. Similarly, the participants were divided into two categories based on their sexual satisfaction using the median as a reference point: (1) individuals with low sexual satisfaction and (2) individuals with high sexual satisfaction.

**Table 1:** Descriptive statistics

Variables	Mean	Median	SD
Relationship satisfaction	5.61	5	1.26
Sexual satisfaction	5.32	5	1.49

Absolute range, 1–7 for both variables

Examining the associations between our grouping variables, chi-square tests of independence showed that the proportions of participants with low, respectively high, relationship satisfaction did not differ by gender, *χ*^2^(1, *N* = 875) = 3.24, *p* = 0.07. Also, there was no significant association between gender and sexual satisfaction, *χ*^2^(1, *N* = 875) = 3.62, *p* = 0.06, or between gender and marital status, *χ*^2^(1, *N* = 875) = 1.01, *p* = 0.32. There were no significant age differences between the two gender groups, *t*(873) = 1.25, *p* = 0.21, but participants with high relationship satisfaction (*M* = 27.89, *SD* = 8.94) were younger than those with low relationship satisfaction (*M* = 31.07, *SD* = 10.62, *t*(873) = 4.39, *p* < 0.01). Similarly, participants with high sexual satisfaction (*M* = 27.02, *SD* = 8.54) were younger than those with low sexual satisfaction (*M* = 31.16, *SD* = 10.56, *t*(873) = 5.65, *p* < 0.01). Relationship satisfaction emerged as unrelated to marital status, *χ*^2^(1, *N* = 875) = 2.33, *p* = 0.13. While the majority of participants in both groups defined by marital status had low sexual satisfaction, this category was significantly more frequent among married participants (81.9% vs. 75% in the unmarried group, *χ*^2^(1, *N* = 875) = 5.59, *p* = 0.02).

### Multiple Correspondence Analysis

**Fig. 1:**
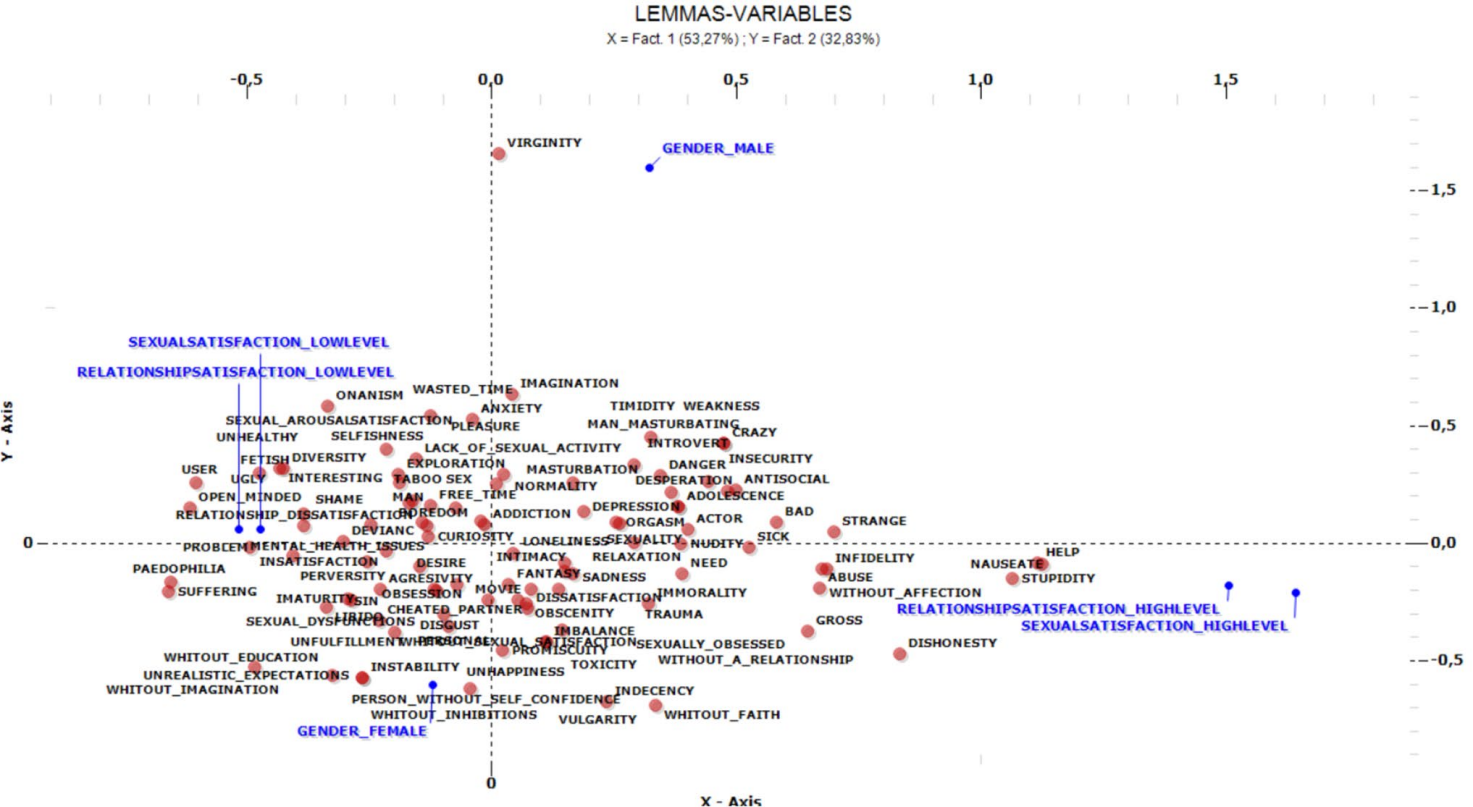
Results of the multiple correspondence analysis

We used multiple correspondence analysis in T-Lab 10.2 (T-LAB Software, 2024) to examine the content of the SR, specifically through the frequency of each of the lexical elements associated by participants with the inductor used (i.e., “pornography user”) and the co-occurrences of these elements. Moreover, this method provides an assessment of the degree to which each lexical element is specific to each of the groups of the variables introduced as independent (or “active”) in the analysis. We used gender, relationship satisfaction, and sexual satisfaction as active variables. The method identifies latent factors that explain the data variability, and represents the co-occurrences between the lexical elements and their relationships to the active variables in a bi-dimensional space (see Fig. 1).

The multiple correspondence analysis extracted two factors, which we will name (1) “perversity–psychological maladjustment” and (2) “lack of confidence and faith–depathologization” to help with interpretation.

Factor 1 (vertical; “perversity–psychological maladjustment”) explains 53.27% of the data inertia (variance). The defining words for Factor 1 (i.e., the lexical elements with the highest t-values) are presented in Table 1.

Factor 2 (horizontal; “lack of confidence and faith–depathologization”) explains 32.83% of the data inertia (variance), and its defining words are presented in Table 2.

**Table 2:** Defining words for Factor 1

Semi-axis negative	*t*-value	Semi-axis positive	*t*-value
Perversity	−2.16	Desperation	1.99
		Dishonesty	2.20
		Help	2.73
		Nauseate	2.75
		Need	2.05
		Sick	2.10
		Strange	2.61
		Stupidity	3.36

Examining the sets of lexical elements that emerged as more closely associated to each of the groups defined by our independent variables, results indicate that men are characterized by common lexical elements, i.e., virginity, man masturbating, normality, desperation, while females are characterized by common lexical elements, i.e., person without self-confidence, without faith. Regarding relationship and sexual satisfaction, individuals with a low level of relationship satisfaction and low level of sexual satisfaction emerged as characterized by common lexical elements such as perversity, while individuals with a high level of relationship satisfaction and high level of sexual satisfaction are characterized by common lexical elements such as dishonesty, stupidity, nauseate, help, strange, sick, and need.

We further examined the specificity of the content of the SR of individuals in each category (low and high levels of sexual and relationship satisfaction, women and men) through the set of typical and exclusive words, respectively, of each of these categories. Examining the set of typical and exclusive words adds a complementary layer to the initial analysis of lexical elements more closely associated with each of the groups. While the first examination identifies general lexical trends and associations within each group, the analysis of typical and exclusive words helps to other pinpoint specific terms that uniquely characterize each group's discourse. The typical lexical elements are those that emerged more frequently in one of the groups, as indicated by a chi-square test comparing their frequencies across categories, while the exclusive elements are those which were associated to the inductor only by participants in one of the groups. The two types of elements pertaining to each gender and level of sexual and relationship satisfaction as active variables are presented in Table 3.

**Table 3:** Defining words for Factor 2

Semi-axis negative	*t*-value	Semi-axis positive	*t*-value
Person without self-confidence	−1.95	Man masturbating	2.13
Without faith	−2.18	Normality	2.03
		Virginity	4.07

## Discussion

This research, building upon the theoretical framework of SRT (Moscovici, 1961, 1987, 1988, 2001), aimed to explore the content of the social representation of the “pornography consumer” within the population of Romania. Our results have highlighted a series of important elements constituting the content of social representation, as well as significant differences based on distinct positioning groups along the psychosocial coordinates considered in this research, i.e., relationship satisfaction, sexual satisfaction, and gender.

First, the characteristic words for men, such as “virginity,” “man masturbating,” “normality,” or “desperation,” imply the “depathologization” of pornography consumption, by involving no critical or stigmatizing perspective on this behavior. The term “normality” may arise due to the well-established fact that men consume more pornography than women (Huțul & Karner-Huțuleac, 2023a; Komlenac & Hochleitner, 2022; Landripet & Štulhofer, 2015; Miller et al., 2019; Sun et al., 2016). Therefore, for them, the use of pornography may fall within the realm of a practice that may be considered routine. Similarly, “man masturbating” and “woman,” which was exclusively evoked by men represent neutral and descriptive association to pornography use, while also restricting this practice to the male gender. The term “virginity” describes a sexually novice or inexperienced individual, which suggests that pornography is represented in this group as a means of acquainting oneself with the subject of sexuality and satisfying one’s sexual needs. The latter use of pornography is also suggested by the term “desperation,” which pinpoints a specific prevailing motivation for pornography consumption, that of providing sexual satisfaction to people who do not have a sexual partner. Besides highlighting these specific functional uses of pornography, both terms may also suggest a nuance of contempt toward those who use it for these purposes, i.e., due to the lack of alternatives, respectively, coping with virginity, as past research found that virginity is often stigmatized among men (Carpenter, 2009; Fleming & Davis, 2018).

Second, results show that the content of women’s SR is centered on “lack of confidence and faith,” implying a stigmatizing and moralizing perspective on pornography consumption. This relates to past findings highlighting that partners’ consumption of pornography may affect women's self-esteem and potentially lowers their self-confidence (Bergner & Bridges, 2002; Daneback et al., 2009; Grov et al., 2011; Perry & Davis, 2017; Schneider, 2000; Stewart & Szymanski, 2012; Zitzman & Butler, 2009). The content of women’s SR, as emerged in our results, puts forward a social understanding of pornography use that condemns consumers and attributes them character flaws that presumably explain their behavior. The reference to religious faith, as well as the term “indecency” that emerged as exclusive to this group, further emphasizes the conservative perspective implied by the content of this SR, sanctioning pornography consumption as violating religious norms and customs, in line with previous findings concerning the relationship between religiosity and adherence to strict moral rules, on the one hand, and the negative perception of pornography, on the other (Grubbs et al., 2015; Huțul & Karner-Huțuleac, 2023a, 2024a; Picone, 2016; Thomas, 2013; Volk et al., 2016).

Among participants with low levels of relationship and sexual satisfaction, the characteristic word was “perversity,” the typical element was “sexual arousal,” and “fetish,” “suffering,” and “diversity” emerged as exclusive words for this group. The terms “perversity” and “fetish” suggest a critical perspective on pornography consumption as a behavior that exaggerates one’s natural sexual drives and turns them in unnatural directions. At the same time, “sexual arousal” and “suffering” suggest a shared understanding of the personal motives of resorting to pornography for satisfying one’s sexual needs when in an unsatisfying relationship. Past research has shown that people resort to the use of pornography as a means of disconnecting from an unsatisfactory sexual life (Peter & Valkenburg, 2010), and the consumption of pornography is associated with couple unsatisfaction (Bridges & Morokoff, 2011; Campbell & Kohut, 2017; Maddox et al., 2011; Muusses et al., 2015; Perry, 2017b; Peter & Valkenburg, 2009). In the same realm, “diversity” suggests the representation of pornography as offering alternative imaginative sexual encounters outside one’s real couple, which pertain to sexual needs that cannot be fulfilled in their romantic relationship. The specificity of this nuance in representing pornography to participants with low relationship and sexual satisfaction further indicates that their primary perspective on pornography consumption is that of adjusting to the shortcomings that they face in their intimate relationships. Thus, these individuals may use pornography as a means of coping with the deficits in their relationship and adjusting their level of sexual satisfaction (Daspe et al., 2018; Olmstead et al., 2013). This representation of pornography use, which lays on the implicit justification of unfulfilled sexual needs, may be a specific facet of a more general positive perspective on engaging in sexual behaviors outside one’s primary relationship, as pornography consumption was found to be associated with one-night stands (Braithwaite et al., 2015), flirtations with other people (Gwinn et al., 2013; Lambert et al., 2012), and infidelity (Wright, 2012).

Participants with high relationship and sexual satisfaction emerged as sharing a SR centered on “dishonesty,” “nauseate,” “help,” “strange,” “sick,” “stupidity,” and “need” as characteristic words. All these terms pertain to a negative and critical view of pornography consumption, with distinct facets. The social understanding of this behavior as stemming from psychological maladjustment is the dominant perspective, as suggested by most of the elements in the list above (i.e., “sick,” “need,” “help,” “strange”). This implies attributing pornography use to psychopathological tendencies, thus exiling it outside of normality and highlighting the need for psychological support for its cure. While words such as “sick,” “nauseate,” or “stupidity” have a negative connotation across contexts, our interpretation of “help” or “need” is based on the semantic contextualization offered by the expressions provided by participants. For instance, “help” was used in negative contexts such as “in need of help,” “specialized help for addicts” or “needs help for his mental problems.” Similarly, “need” was used by expressions such as “needs a doctor,” “needs to see mental health professionals,” or “needs a real, not imaginary, sex life.” Secondly, the emergence of the term “stupidity” as characteristic for this group may suggest that individuals who are satisfied with their relationship consider pornography consumption as lacking any rational motivation, while also having potential risks. The latter nuance may relate to actual research findings, showing that pornography use can lead to conflicts, instability in the relationship (Poulsen et al., 2013), and may diminish sexual satisfaction or reduce sexual attraction to the partner (Albright, 2008; Manning, 2006). Thus, when the couple relationship is satisfying, pornography viewing is perceived as a potential enemy of the relationship itself.

Finally, “dishonesty” and “nauseate” not only accentuate the negative view on pornography, but also put forth the moral dimension as another reference line for this negative evaluation (“immorality” also emerged as a typical word for the high relationship satisfaction group). This area of SR content highlights a conservative view of pornography as violating societal and moral norms, and its emergence in this category of participants may indicate that people satisfied in their romantic relationships are more likely to apply the conventional lenses of understanding pornography consumption, with no inclination to consider the potential personal or relational motives that may foster it. Contrarily, this behavior is mostly framed as morally sanctionable, while from a pragmatic point of view, it is not only useless but also dangerous, thus justifying the portrayal of people who engage in such behaviors as psychologically maladjusted.

In sum, our findings indicate that the social representation of pornography consumers varies significantly depending on gender, sexual satisfaction, and relationship satisfaction. Women and people satisfied by their romantic relationship share a conservative, critical and moralizing view on this matter. While women focus on alleged character flaws that drive pornography consumers toward this behavior, individuals with high sexual and relationship satisfaction emphasize its irrationality and dangers, describing these consumers as psychologically maladjusted. Individuals less satisfied with their romantic relationship partly share the critical view on pornography use, while also emphasizing its potential of offering a means to sexually adapt to the limitations and frustrations in one’s relationship. Finally, the dominant perspective in the male group emerged as focused on specific contextual motivations for pornography use, i.e., one’s lack of sexual experience and/or partner. Since men are the primary consumers of pornography (e.g., Miller et al., 2019), this somewhat neutral understanding of pornography use, with no attribution of blame, mental illness or personality flaws does not highlight any apparent risks in terms of the self-stigmatization of consumers. Nevertheless, when considering both gender and the dimension of relationship and sexual satisfaction, our results suggest that males who tend to be unsatisfied in their relationship are prone to perceive pornography use in more critical terms, as a mark or factor of an unnatural sexual drive. At the same time, they also acknowledge the potential role of pornography consumption in satisfying one’s otherwise frustrated sexual needs. In the long run, this psychological conflict can put these individuals at risk for self-stigma, guilt and consequent mental health consequences (Grubbs et al., 2019; Tewksbury, 2012), challenging to manage if adaptive coping mechanisms to deal with the resulting stress are lacking. Furthermore, the other two categories considered by our research (i.e., women and individuals with high relationship satisfaction) share a negative and judgmental view on pornography consumption, with no empathic concern for one’s eventual personal and/or contextual motives that may justify this behavior. This can create the risk of social stigmatizing pornography consumers, thus exacerbating the psychological conflicts faced by individuals who are unsatisfied in their romantic relationship in relation to pornography use.

The content of SRs varies across cultures; therefore, our findings are relevant for the Romanian context and cannot be readily generalized to other cultural settings. As previously highlighted, pornography use is considered a morally condemnable subject in Romania, a country in which the Christian Orthodox plays a crucial role in shaping the values held by its population (Gergely & Rusu, 2023; Huțul & Karner-Huțuleac, 2023a; Turcescu & Stan, 2005). Thus, the social stigma attached to pornography could be more pronounced than those found in less conservative cultures. Other contextual factors may also be important in creating significant cross-cultural variations in the SR of pornography. Furthermore, it is important to consider that our sample consisted exclusively of participants currently in a relationship; hence, our findings do not pertain to the SR of individuals who are not engaged in a committed relationship.

### Theoretical and Practical Implications

From a theoretical standpoint, our study contributes to the conceptualization of social representations theory in the realm of pornography consumption (Moscovici, 1961, 1987, 1988, 2001). Exploring the content of these representations enhances understanding of the cultural impact of pornography, identifying prevalent perceptions and attitudes regarding pornography within social contexts. Moreover, this enhanced knowledge can improve theoretical understanding of the outcomes of pornography use. These outcomes of pornography use can be shaped by context into psychological distress or positive outcomes, based on individual differences. In the framework of the PPMI model (Grubbs et al., 2019), our findings pinpoint a distinct potential source of the moral incongruence between one’s moral beliefs sanctioning pornography use and one’s actual consumption behavior, specifically the negative and judgmental SRs that the individual may hold. These may foster the moral disapproval of one’s behavior and, consequently, moral incongruence and psychological conflict. Pornography users may also be at risk of distress due to the negative SRs on this topic that are shared and expressed by individuals who are personally significant to them, such as family and friends, which can shift the moral appraisals of their own behavior toward disapproval and therefore induce internal conflicts (Table 4)

**Table 4:** Typical and exclusive words based on relationship and sexual satisfaction, and gender

High level of relationship satisfaction				Low level of relationship satisfaction			
Typical words	χ^2^	*p*	Exclusive words	Typical words	χ^2^	*p*	Exclusive words
Stupidity	14.95	0.001		Sexual arousal	4.26	0.03	Fetish
Help	10.1	0.001					Suffering
Need	5.93	0.01					Diversity
Nauseate	5.06	0.02					
Immorality	4.67	0.03					
Strange	4.08	0.04					

High level of sexual satisfaction				Low level of sexual satisfaction			
Typical words	χ^2^	*p*	Exclusive words	Typical words	χ^2^	*p*	Exclusive words
Dishonesty	9.54	0.002		Curiosity	4.86	0.02	Problem
Nauseate	6.65	0.009		Unsatisfaction	4.52	0.03	Suffering
Strange	6.02	0.01		Perversity	3.88	0.04	Interesting
Infidelity	4.77	0.02					
Stupidity	4.29	0.03					
Sick	4.1	0.04					

Males				Females			
Typical words	χ^2^	*p*	Exclusive words	Typical words	χ^2^	*p*	Exclusive words
Man masturbating	5.56	0.01	Virginity	–			Person without self-confidence
Normality	3.93	0.04	Woman				Without faith
							Indecency

From a practical standpoint, our study can serve as a cornerstone for the development of therapeutic intervention plans used by clinicians, psychotherapists, and mental health workers who encounter issues related to pornography consumption in their practice. By considering the dominant SRs in the social categories to which their patients belong, as well as their level of sexual and relationship satisfaction, mental health specialists can orient their therapeutic interventions accordingly. At the same time, conclusions drawn from the content of social representations of pornography consumers can be integrated into training programs for future mental health professionals. Consequently, they can be better equipped to address issues related to pornography consumption in clinical settings.

### Limitations and Future Directions

Despite the positive aspects of our study, we must address a series of limitations. Firstly, the use of pornography is a taboo subject in Romania (Gergely & Rusu, 2023; Huțul & Karner-Huțuleac, 2023a), and participants' responses may be shaped around social desirability. Secondly, our study involved a significantly lower proportion of men than women, limiting the generalizability of our results, as men consume more pornography than women and may be more likely to have positive attitudes toward it (Huțul & Karner-Huțuleac, 2023a; Komlenac & Hochleitner, 2022; Landripet & Štulhofer, 2015; Miller et al., 2019; Sun et al., 2016). Future studies should take this into account and attempt to balance the gender distribution. Thirdly, another limit of our study is that we did not control for the variations in the content of the SR associated to age, as SRs of pornography of younger and older adults may differ significantly. Future works employing qualitative approaches should consider this aspect and examine, for instance, how age shapes the SR of pornography alongside factors pertaining to the quality of one’s romantic relationship, as our results indicate negative relationships between age and relationship and sexual satisfaction, respectively. Fourthly, all our instruments were of the self-report type, and the categorization of participants in high vs. low relationship and sexual satisfaction was performed through the use of the median, an indicator that depends on the distribution of the scores in the study sample. Further research should employ more psychometrically sound approaches in classifying participants in the groups of interest. Fourthly, our study did not include a measure of participants' use of pornography, which may be an important factor in their SRs. Future investigations on the social understanding of pornography could use an indicator of this behavior, such as the approximate number of hours people spend watching pornography weekly. Furthermore, they could target other objects of social representation that may be relevant for people’s consumption of pornography and its associated mental and couple dynamics, such as romantic or sexual relationships.

## Data Availability

The data are available upon request at any time to the corresponding author.
